# Comparison of [^18^F]DOPA and [^68^Ga]DOTA-TOC as a PET imaging tracer before peptide receptor radionuclide therapy

**DOI:** 10.1186/s41824-022-00133-6

**Published:** 2022-06-15

**Authors:** Emile B. Veenstra, Adrienne H. Brouwers, Derk Jan A. de Groot, Johannes Hofland, Annemiek M. E. Walenkamp, Tessa Brabander, Wouter T. Zandee, Walter Noordzij

**Affiliations:** 1 Medical Imaging Center, Department of Nuclear Medicine and Molecular Imaging, University of Groningen, University Medical Center Groningen, P.O. Box 30.001, 9700 RB Groningen, The Netherlands; 2 Department of Medical Oncology, University of Groningen, University Medical Center Groningen, Groningen, The Netherlands; 3 Department of Internal Medicine, Section of Endocrinology, Erasmus University Medical Center, Rotterdam, The Netherlands; 4 Department of Nuclear Medicine and Molecular Imaging, Erasmus University Medical Center, Rotterdam, The Netherlands; 5 Department of Internal Medicine, Section of Endocrinology, University of Groningen, University Medical Center Groningen, Groningen, The Netherlands

**Keywords:** Neuroendocrine tumour, [^18^F]DOPA, [^68^Ga]DOTA-TOC, [^177^Lu]DOTA-TATE, Peptide receptor radionuclide therapy, PRRT

## Abstract

**Background:**

In treatment of neuroendocrine neoplasms (NENs), confirmation of somatostatin receptor expression with ^68^Ga-DOTA somatostatin analogues is mandatory to determine eligibility for peptide receptor radionuclide therapy (PRRT). [^18^F]DOPA can detect additional lesions compared to [^68^Ga]DOTA-TOC. The aim of this study was to explore differences in tumour detection of both tracers and their relevance for selecting patients for PRRT. We retrospectively studied eight patients with NENs who underwent both [^68^Ga]DOTA-TOC and carbidopa-enhanced [^18^F]DOPA PET/CT, before first-time PRRT with [^177^Lu]DOTA-TATE. Tracer order was influenced due to stock availability or to detect suspected metastases with a second tracer. On CT, disease control was defined as a lesion showing complete response, partial response, or stable disease, according to RECIST 1.1. criteria.

**Results:**

Seven patients with in total 89 lesions completed four infusions of 7.4 GBq [^177^Lu]DOTA-TATE, one patient received only two cycles. Before treatment, [^18^F]DOPA PET/CT detected significantly more lesions than [^68^Ga]DOTA-TOC PET/CT (79 vs. 62, *p* < .001). After treatment, no difference in number of lesions with disease control was found for [^18^F]DOPA-only (5/27) and [^68^Ga]DOTA-TOC-only lesions (4/10, *p* = .25). [^18^F]DOPA detected more liver metastases (24/27) compared to [^68^Ga]DOTA-TOC (7/10, *p* = .006). Six patients showed inpatient heterogeneity in treatment response between [^18^F]DOPA-only and [^68^Ga]DOTA-TOC-only lesions.

**Conclusions:**

Response to PRRT with [^177^Lu]DOTA-TATE was comparable for both [^68^Ga]DOTA-TOC- and [^18^F]DOPA-only NEN lesions. [^18^F]DOPA may be capable of predicting response to PRRT while finding more lesions compared to [^68^Ga]DOTA-TOC, although these additional lesions are often small of size and undetected by diagnostic CT.

## Background

Neuroendocrine neoplasms (NENs) are a group of heterogeneous tumours with both neural and endocrine histological features. Neuroendocrine cells are typically regulated by numerous hormones, acting through specific receptors on the membrane surface. An overexpression of these receptors in most gastrointestinal NENs poses the basis for current functional PET/CT imaging and peptide receptor radionuclide therapy (PRRT) (Bodei et al. 2009).

PRRT using the somatostatin agonist tyrosine3-octreotate (DOTA-TATE) is an accepted treatment option for metastatic and non-resectable somatostatin receptor-positive NENs. Confirmation of receptor affinity, with ^68^Ga-labelled somatostatin analogues (SSAs) diagnostic imaging, is mandatory before determining a patient’s eligibility for PRRT (Kwekkeboom et al. 2010). Three ^68^Ga-labelled SSA tracers are commonly used: DOTA-TATE, -TOC, and -NOC. The widespread introduction of PRRT for NEN was stimulated by the completion of the phase III NETTER-1 study (Strosberg et al. 2017). PRRT with ^177^Lu-labelled SSAs demonstrated treatment efficacy, with disease control rates ranging between 80 and 90% (Bodei et al. 2016; Kim et al. 2015; Brabander et al. 2017).

In clinical practice, more intense tumour ^68^Ga-labelled SSA uptake compared to normal liver parenchyma determines a patient’s suitability for PRRT. However, somatostatin receptor (SSTR) distribution and density are sometimes limited (Wild et al. 2013; Sundin et al. 2007). For well-differentiated NEN tumours, another widely studied tracer is 6-l-[^18^F]fluoro-dihydroxyphenylalanine ([^18^F]DOPA). [^18^F]DOPA provides information about the dopamine pathway of tumour cells, which is often increased in NENs. The main clinical use for NEN imaging with [^18^F]DOPA is for midgut and hindgut NEN, certain paragangliomas, and NEN with low or variable SSTR expression (Bozkurt et al. 2017). [^18^F]DOPA offers increased image quality compared to [^68^Ga]DOTA-TOC, excelling in locating small intestinal tumours (Ambrosini et al. 2015). Although studies detailing the complex relation between glucose metabolism and SSTR expression exist (Oh et al. 2011), no studies have explored potential relationships between the dopamine pathway and SSTR presence.

As [^18^F]DOPA and [^68^Ga]DOTA-TOC utilize different aspects of tumour biology (Ambrosini et al. 2015), [^18^F]DOPA may identify lesions in tumours with weak or absent expression of SSTR. It is yet unclear if [^18^F]DOPA can be a substitute of [^68^Ga]DOTA-TOC in selecting PRRT candidates. We hypothesize that [^18^F]DOPA is equally capable in predicting response to PRRT, compared to [^68^Ga]DOTA-TOC. Therefore, in this study, we studied the lesion-based relationship between pre-treatment visualization on [^18^F]DOPA and [^68^Ga]DOTA-TOC PET/CT, and response to PRRT treatment.

## Methods

### Patients and study design

Eight patients who underwent both [^68^Ga]DOTA-TOC and [^18^F]DOPA PET/CT scanning before treatment with [^177^Lu]DOTA-TATE between May 2015 and February 2019 were retrospectively included in this case series. Pre- and post-treatment PET and CT scans were performed in the University Medical Center Groningen, while PRRT was performed in the Erasmus Medical Center. All included patients had histologically proven NEN before their first-time treatment with PRRT. Median follow-up was 41 months (interquartile range [IQR]: 24–54) after first cycle of PRRT. Tumour grade (according to the 2010 World Health Organization classification) and therapy changes during follow-up were retrieved from the electronic patient chart (Kim et al. 2017).

Before PRRT, patients were confirmed, by [^68^Ga]DOTA-TOC PET/CT, to have sufficient SSTR presence in target lesions. Lesions were found to be sufficiently SSTR-active if [^68^Ga]DOTA-TOC uptake was higher than the liver. Additionally, due to suspicion of non-[^68^Ga]DOTA-TOC avid lesions or for logistical reasons, patients also received [^18^F]DOPA PET/CT. Suspicion might be due to finding hypodense [68Ga]DOTA-TOC non-avid lesion on diagnostic CT or due to mismatch of [68Ga]DOTA-TOC lesion appearance and clinical presentation. No [^18^F]FDG PET scans were performed, as there was no suspicion for G3 NET or NEC in the included patients.

### [^68^Ga]DOTA-TOC and [^18^F]DOPA PET/CT

[^68^Ga]DOTA-TOC was produced using a Scintomics module and an Eckert and Ziegler [^68^Ge]/[^68^Ga] generator (Schultz et al. 2013). [^18^F]DOPA synthesis was performed as previously reported (Luurtsema et al. 2017). Imaging for both tracers was realized by a Siemens Biograph mCT (40 or 64 slices, 4 detector rings, Siemens Healthcare, Knoxville, TN, USA). Attenuation and scatter correction of the PET emission data was achieved by a low-dose CT scan with 120 kV and 35 mAs. Image reconstructions were obtained using three-dimensional ordinary Poisson ordered-subset expectation maximization with application of Time-of-Flight, three iterations, 21 subsets, and a Gaussian filter of 5 mm. The resulting image size was 400 × 400 with a voxel size of 2 × 204 × 204 mm. . Contrast-enhanced CT scans of the chest and abdomen at baseline and response evaluation were obtained by using iodine-containing intravenous iomeprol (Iomeron) 350 mg/mL.

Patients were hydrated with 1 L of water before acquisition of both scans. Sixty minutes before the injection of [^18^F]DOPA, patients were pre-treated with carbidopa (2 mg/kg body weight, max. 150 mg). Patients received an intravenously injected dose of 200 megabecquerel (MBq) [^18^F]DOPA, or 120 MBq [^68^Ga]DOTA-TOC. Images were acquired after 60 ± 5 min, scanning from mid-thigh to head. [^18^F]DOPA PET/CT was acquired in 1.5 min per bed position in patients less than 60 kg body weight, 2 min per bed position in patients weighing 60–90 kg body weight and 3 min per bed position in patients over 90 kg body weight. [^68^ Ga]DOTA-TOC PET/CT was acquired from proximal femur to head, with 3 min per bed position, irrespective of body weight.

### ^177^Lutetium treatment

A maximum of four cycles of 7.4 GBq of [^177^Lu]DOTA-TATE was intravenously infused over a timeframe of 30 min. Patients received infusions every 6 to 12 weeks (goal cumulative activity: 29.6 GBq). Preparation and administration protocol was described earlier (Zandee et al. 2019).

### Image data analysis and statistics

Scans were analysed with Syngo.via (version VB30, Siemens Healthcare, Germany). Contrast-enhanced CT performed before and after PRRT was used to determine treatment effects and aided in verification of lesion malignancy together with radiological or histopathological reports. On PET/CT, a lesion was positive if a clear demarcated lesion could not be attributed to physiological uptake patterns. Results of both PET tracers were used to make lesion comparisons. On CT, longest axis diameters of all lesions were determined on the transverse plane. All individual lesions found by any PET tracer were cross-verified by their respective presentation on the CT scans, if any. Resulting CT-positive tumours were then individually labelled by their respective RECIST 1.1 categories: complete response (CR), partial response (PR), stable disease (SD), and progressive disease (PD). An additional category named ‘undetermined’ was created for positive PET/CT lesions with no substrate presence on CT. RECIST evaluation was not possible for these undetermined lesions. Disease control was present if a lesion was determined to be CR, PR, or SD.

Matching lesions were lesions that were seen by both tracers. A patient contained a mismatch pattern by having at least one lesion not detected by both tracers, while other lesions were concordant. Heterogeneity in treatment response occurred if a single patient had DOTA-TOC-only or matched lesions with at least one lesion with disease control (CR/PR/SD) and one without (PD).

Statistical analysis was performed using SPSS 23.0 software (SPSS Inc., Chicago, IL). Non-normally distributed outcomes were reported by medians and interquartile ranges. Chi-squared test was performed on all comparisons between [^18^F]DOPA- and [^68^Ga]DOTA-TOC-only lesions and Fisher’s exact test on RECIST score and tracer uptake subgroup (DOTA-TOC-only, DOPA-only, matched lesions). P values lower than 0.05 were considered to be significant.

## Results

### Patients

Eight patients (5 male, 3 female) with median age of 69 (IQR: 65–71) years old were included (Table 1). Seven patients had well-differentiated NENs (two G1, five G2), but for one patient, no tumour grading could be found. Primary tumour location was small intestine in five patients, colon in two, and lung in one. Before PRRT, ileo-caecal resection was performed in three patients, jejunal resection in one, and hemicolectomy in one. One patient had received everolimus and one patient interferon-gamma immunotherapy and telotristat for carcinoid syndrome. During PRRT, patients continued treatment with SSAs. All adverse events were self-limiting: two patients had minor gastrointestinal issues, two mild thrombocytopenia, two mild anaemia, and one mild neutropenia. Seven patients completed four infusions of 7.4 GBq [^177^Lu]DOTA-TATE, and one patient could not continue treatment because of progressive disease after two cycles.

**Table 1:** Patient characteristics

No	Age (years)	Sex	Primary tumour	Tumour grade (Ki-67)	Prior therapies	Current therapies	Diagnosis to start PRRT (months)	Time between [^18^F]DOPA and [^68^Ga]DOTA-TOC (months)	Time between PRRT-end and death (months)	PRRT cycles	Cumulative activity administered by PRRT (GBq)	Time between last PET/CT and start PRRT (months)	Pre-CT to PRRT^a^ (months)	PRRT to Post-CT^b^ (months)
1	55	F	SI	G2 (2.8%)	Everolimus	Lanreotide	11	5	–	4	30.1	5	0	2
2	70	M	Colon	G2 (3.5%)	Surgery, interferon alpha, telotristat	Lanreotide, Octreotide SA	37	13	–	4	29.9	9	2	2
3	58	F	SI	G2 (10%)	Surgery	Octreotide SA	39	1	5	2	14.9	5	2	2
4	74	M	SI	G1 (< 2%)		Sandostatine	97	1	–	4	30.1	5	5	11
5	80	M	SI	G2 (< 2%)	Surgery	Sandostatine	181	1	–	4	29.6	2	3	2
6	67	M	SI	G2 (< 2%)	Surgery	Lanreotide, Octreotide SA	107	1	–	4	28.7	3	4	6
7	69	F	Colon	G1 (< 2%)	Surgery, SSA	Sandostatine,	38	1	–	4	29.1	2	3	4
8	70	M	Lung	N/F (< 2%)		Octreotide SA	36	5	27	4	29.7	3	3	2

*F*, female; *M*, male; *SI*, small intestine; *N/F*, not found

^a^Days between last performed CT before PRRT, and first PRRT-cycle, ^b^days between last PRRT-cycle and first CT performed after PRRT

### Tumours

In total, more lesions were visualized with [^18^F]DOPA PET/CT compared to [^68^Ga]DOTA-TOC PET/CT (79 vs. 62, *p* = 0.00, Table 2). Combining the results of both PET tracers counted 89 total and 52 matching lesions. Twenty-seven lesions were only found on [^18^F]DOPA PET/CT and 10 lesions were only found on [^68^Ga]DOTA-TOC PET/CT (Table 3). [^18^F]DOPA-only detected more liver metastases (24/27, 2 PR, 1 SD, 4 PD, 17 UD) compared to [^68^Ga]DOTA-TOC-only (7/10, 2 PR, 1 SD, 3 PD, 1 UD, *p* = 0.006). Three mediastinal lesions were DOTA-TOC-only as well (1 PR, 2 UD).

**Table 2:** Response per patient, as detected on [18F]DOPA and [68Ga]DOTA-TOC PET/CT

No	TTP^a^ (months)	TTD^b^ (months)	RECIST (1-year)	Total lesions (n = 89)	Total matched lesions (n = 52)	Total DOPA lesions (n = 79)	DOPA-only lesions (n = 27)	DOPA-only tumour size (mm, mean)	Total DOTA lesions (n = 62)	DOTA-TOC-only lesions (n = 10)	DOTA-only tumour size (mm, mean)
1	–	–	PR	10	8	10	2	42	8	0	40
2	18	–	PR	7	3	14	11	19	3	0	14
3	0	5	PD	8	7	8	1	27	7	0	25
4	–	–	SD	10	4	4	0	18	10	6	31
5	–	–	SD	18	6	17	11	25	7	1	28
6	15	–	SD	14	7	7	0	16	7	0	16
7	14	–	SD	7	6	7	1	23	6	0	24
8	7	27	PD	15	11	12	1	14	14	3	41

*PR*, Partial response; *PD*, progressive disease; *SD*, stable disease

^a^Time to progression, ^b^time to death (counting from date of last cycle of PRRT)

**Table 3:** Response per lesion location as detected on [18F]DOPA and [68 Ga]DOTA-TOC PET/CT ^a^

	Total number of lesions	Partial response	Stable disease	Progressive disease	Undetermined
*DOPA tracer only*					
Total	27	3	2	4	18^*^
Liver	24^*^	2	1	4	17
Mesentery	2	1	1	–	–
Cardiac	1	–	–	–	1
*DOTA tracer only*					
Total	10	3	1	3	3^*^
Liver	7^*^	2	1	3	1
Mediastinum	3	1	–	–	2
*DOPA + DOTA tracer*					
Total	52	24	7	7	14
Liver	22	10	4	3	5
Bone	8	–	2	2	4
Mesentery	8	6	1	1	–
Cardiac	4	–	–	1	3
Lymph nodes	6	2	0	3	1
Pancreas	2	2	–	–	–
Lung	2	1	–	–	1

^a^No tumours with complete response were found

^*^Significant difference found between tracers (*p* < .05)

Forty-five lesions (out of 89) were detected on pre- and post-PRRT CT, of which nine DOPA-only (3 PR, 2 SD, 4 PD), seven DOTA-TOC-only (3 PR, 1 SD, 3 PD), and 38 matching lesions (24 PR, 7 SD, 7 PD). Any difference found between lesion tracer uptake subgroup category and RECIST score was non-significant (*p* = 0.316). Number of lesions showing disease control was not significantly different between DOPA-only (5/27) and DOTA-TOC-only lesions (4/10, *p* = 0.25) and between all lesions found by both (*p* = 0.505). Matching lesions were mostly in the liver (22/52, 10 PR, 4 SD, 3 PR, 5 UD). The remaining 30 matching lesions consisted of eight bone, eight mesenteric, six cardiac, four lymph, two pancreas, and two lung lesions. Seven out of eight patients showed mismatch results between tracer uptake on a lesion-based analysis. Mismatch results were located in the liver (32/37), mesenteric (2/37), and myocardial, mediastinal, paratracheal, and substernal (all had one).

**Fig. 1:**
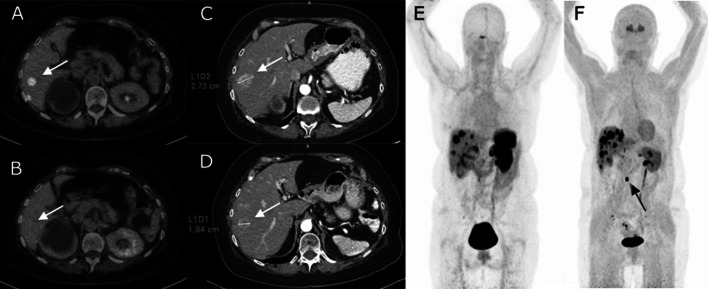
Imaging of a 70-year-old female patient (#2) with a metastasized primary colon NEN (G2). Fourteen tumours were found, of which 11 only by [18F]DOPA PET/CT and three by both PET tracers. PET/CT shows tumour uptake of [18F]DOPA in the liver (**A**), while [68 Ga]DOTA-TOC shows none (**B**). The same tumour had a longest axis diameter of 27 mm on the pre-PRRT CT scan (**C**), reduced to 18 mm in the post-PRRT CT scan (**D**). These images display significant therapy effect of PRRT in a tumour only found by [18F]DOPA. Maximum intensity projection (MIP) images show higher incidence of focal uptake of [18F]DOPA (**F**) in the liver compared to [68 Ga]DOTA-TOC (**E**). A positive para-aortal lymph node was found only by [18F]DOPA (F, black arrow)

In six patients, heterogeneity in treatment response was observed. Patient 1 showed treatment effect in several matching liver tumours, while a matched thoracic spinal lesion showed progression. The same event occurred in DOTA-TOC-only tumours: patient 4 had one liver lesion showing PR, while several other liver lesions being PD. DOPA-only lesions showed this effect as well: in patient 2, one liver lesion showed treatment response, while another showed progression (Fig. 1).

### Follow-up

The one-year radiological response of the lesions (RECIST 1.1) was PR in two patients, SD in four, and PD in two. One patient had progressive disease after seven months, two patients after 15 months, and one patient after 18 months of follow-up. Two patients died during follow-up. One patient dropped out after the second cycle of PRRT with on-going progressive disease and died five months afterwards. Another patient died after 27 months follow-up. Both deaths were due to end-stage metastatic liver disease.

## Discussion

Our study showed that [^18^F]DOPA found a higher number of lesions compared to [^68^Ga]DOTA-TOC. Generally, response to PRRT with [177Lu]DOTA-TATE was comparably predicted for both [^68^Ga]DOTA-TOC- and [^18^F]DOPA-only NEN lesions. While potentially incidental, we do note that of the 27 lesions visible with [^18^F]DOPA, three showed PR and four PD, which could suggest that non-[^68^Ga]DOTA-TOC avid lesions can respond to PRRT with [^177^Lu]DOTA-TATE. Both matching and mismatch lesions were predominantly found in the liver. In six patients, heterogeneity in treatment response was noted in both DOPA-only as DOTA-only lesions. In this small cohort, treatment with PRRT was generally effective at one-year follow-up, as six out of eight patients had disease control and two had progressive disease.

SSTR subtype targeting differences between diagnostic DOTA-TOC and therapeutic DOTA-TATE could explain conflicting results in found and responding lesions. Five SSTR subtypes have been identified, expressed in differing frequencies in NENs. As the majority of NENs express SSTR2 and 5, the use of PET/CT with ^68^Ga-labelled SSAs to determine eligibility has been the logical choice since the deployment of PRRT (Wild et al. 2013; Kwekkeboom and Krenning 2016). Several SSA PET tracers have consequently developed, all with different SSTR subtype targeting profiles. [^68^Ga]DOTA-TATE shows high affinity for SSTR2, [^68^Ga]DOTA-TOC with SSTR2 and 5 (Bozkurt et al. 2017). Potentially, even better lesion detection rates could be achieved using [^68^Ga]DOTA-NOC, displaying the highest affinity with SSTR2 and 5 (Wild et al. 2013). Additionally, SSTR antagonists are promising for imaging and treatment, due to more stable binding and expressing practically all SSTR subtypes, but currently lack large clinical trials (Bozkurt et al. 2017; Ginj et al. 2006; Minczeles et al. 2021).

SSTR heterogeneity of [^68^Ga]DOTA-TOC within-tumour uptake is associated with worse response to PRRT (Graf et al. 2020), it is yet unknown if within-patient tracer uptake heterogeneity carries any prognostic value of response to PRRT. Our patients demonstrated one-sided heterogeneity of tracer uptake: only two out eight patients had both DOPA-only and DOTA-TOC-only lesions. This is further expanded by the finding that both DOPA-only and DOTA-TOC-only lesions showed heterogeneity in treatment response for six out of eight patients. Our study found four out of ten DOTA-TOC-only lesions showing treatment effect compared to four out of nine DOPA-only lesions. This challenges the notion that ^68^Ga-labelled SSAs positive and [^18^F]DOPA negative lesions were more likely to respond to PRRT (Ambrosini et al. 2008). Our results show that DOTA-avid lesions can be dissimilarly affected by PRRT, and that DOPA-only lesions show treatment effect.

[^18^F]DOPA is often used in low-SSTR NENs, although availability in clinical centres is usually less than DOTA. Discrepancies in tumour detection between [^18^F]DOPA and [^68^Ga]DOTA-TOC exist, as both tracers utilize different receptor pathways (Ambrosini et al. 2015). [^18^F]DOPA identified many liver metastases that were not detected by [^68^Ga]DOTA-TOC. Most of these [^18^F]DOPA-only tumours were not seen on pre- and post-PRRT CT scans due to their small size (17/27). Although outside the scope of our study, we hypothesize that therapy response for undetermined [^18^F]DOPA-only lesions could be established by dynamic contrast-enhanced MRI or PET/MRI. These small and often diffuse DOPA-only lesions in the liver could be a sign of new, and potentially poorly differentiated, metastasis of NEN. This is in accordance with findings that [^18^F]DOPA PET/CT has been shown to more accurately evaluate small lesions with low-SSTR expression, such as poorly differentiated tumours (Ambrosini et al. 2008). Our finding that more lesions were found by [^18^F]DOPA compared to [^68^Ga]DOTA-TOC PET/CT are in contrast with previous comparative studies, which we commented on in our previous study (Veenstra et al. 2021). We cannot make a recommendations on dual-tracer imaging with [^18^F]DOPA and [^68^Ga]DOTA on patients before PRRT, based on small study population. In the case of non-DOTA-avid lesions, [^18^F]DOPA might be considered as additional tracer, especially in G1 and G2 NEN. For selected cases, PRRT in G3 NEN is a developing and promising field of study and here dual-tracer imaging with [^18^F]FDG would be appropriate instead of [^18^F]DOPA.

Our study was limited by its retrospective scope and dropout of one patient, only finishing two cycles. Determining any effects of PRRT in this patient was exceedingly difficult, due to severe ascites present in post-PRRT CT scans. Several outliers in scan characteristics were apparent. One patient had large time frame between PET/CT scans (384 days, other patients 180) and PET/CT to PRRT (270 days, other patients < 170). Another patient reported a 330-day delay between PRRT and post-CT (other patients 180). Five patients had a follow-up CT at 2 months after completion of all PRRT cycles, although uncommon, tumour pseudo progression due to radiation-induced oedema can occur which could have influenced the results of this study.

[^68^Ga]DOTA-TOC or [^68^Ga]DOTA-TATE PET/CT are the current diagnostic modalities to select patients for PRRT. In our case series, [^18^F]DOPA PET/CT showed more tumours compared to [^68^Ga]DOTA-TOC PET/CT. Response to PRRT was comparable for both DOTA- and DOPA-only lesions, with [^18^F]DOPA finding more liver metastases. [^18^F]DOPA may be capable of predicting response to PRRT and can detect tumour receptor heterogeneity. Further prospective studies with controlled scan intervals and randomized tracer allocation are needed to improve clinical knowledge of [^18^F]DOPA in selecting and follow-up of PRRT in patients with NENs.

## Data Availability

The datasets used and/or analysed during the current study are available from the corresponding author on reasonable request.

## Abbreviations

PRRT: Peptide receptor radionuclide therapy
DOTA-TATE: DOTA-(Tyrosine)-octreotate
[^18^F]DOPA: 6-L-[^18^F]fluoro-dihydroxyphenylalanine
RECIST: Response evaluation criteria
SSAs: Somatostatin analogues
SSTR: Somatostatin receptor
CR: Complete response
PR: Partial response
SD: Stable disease
PD: Progressive disease
